# AI-Powered Preoperative Chest Radiograph-Derived Cardiovascular Border Indices and Risk of Postoperative Major Adverse Cardiovascular Events: A Large-Scale Retrospective Cohort Study

**DOI:** 10.3390/medicina62101897

**Published:** 2026-09-30

**Authors:** Hwa-Young Jang, Jeong Hwan Kim, Hong Min Oh, Hong-Cheol Yoon, Chang-Woo Kim, Woo-Jin Kim, Hyun-Seok Kim, Woo-Young Seo, Dong Hyun Yang, Sung-Hoon Kim

**Affiliations:** 1 Department of Anesthesiology and Pain Medicine, Asan Medical Center, Brain Korea 21 Project, University of Ulsan College of Medicine, 88 Olympic-ro 43-gil, Songpa-gu, Seoul 05505, Republic of Korea; 2 Department of Radiology (Cardiovascular Imaging), Asan Medical Center, University of Ulsan College of Medicine, 88 Olympic-ro 43-gil, Songpa-gu, Seoul 05505, Republic of Korea; 3 Big Data Research Center, Asan Institute for Life Science, Asan Medical Center, 88 Olympic-ro 43-gil, Songpa-gu, Seoul 05505, Republic of Korea; 4 Biomedical Engineering Research Center, Asan Institute for Life Science, Asan Medical Center, 88 Olympic-ro 43-gil, Songpa-gu, Seoul 05505, Republic of Korea; 5 Institute of Digital Healthcare, University of Ulsan College of Medicine, 88 Olympic-ro 43-gil, Songpa-gu, Seoul 05505, Republic of Korea

**Keywords:** radiography, thoracic, artificial intelligence, perioperative care, postoperative complications, risk assessment

## Abstract

*Background and Objectives*: Routine preoperative chest radiography (CXR) has limited value for perioperative risk prediction when interpreted qualitatively. The Automated Diagnosis of Cardiovascular abnormalities (ADC) model enables automated quantification of cardiomediastinal vascular border (CVB) parameters on CXR. This study evaluated the associations of AI-derived CVB metrics with postoperative major adverse cardiovascular events (MACE) and their incremental predictive value beyond clinical factors. *Materials and Methods*: This retrospective cohort study included patients who underwent surgery under general anesthesia at a tertiary academic center. The ADC model quantified CVB parameters as raw measurements and age- and sex-adjusted z-scores. The primary outcome was postoperative in-hospital MACE. Associations were assessed using multivariable logistic regression, and discrimination was evaluated using receiver operating characteristic curve analysis. Incremental predictive performance was assessed by adding selected CVB parameters to a clinical reference model. *Results*: Among 101,531 patients, MACE occurred in 1655 patients (1.6%). All ADC-derived CVB parameters remained independently associated with MACE after multivariable adjustment. For cardiothoracic ratio, the adjusted OR was 1.64 (95% CI, 1.58–1.70; *p* < 0.001), with an AUC of 0.789. For the composite CVB z-score, the adjusted OR was 1.88 (95% CI, 1.79–1.97; *p* < 0.001), with an AUC of 0.762. Adding either the CT ratio or composite CVB z-score to the clinical reference model increased the AUC from 0.894 to 0.906 (*p* < 0.001 for both), with similar improvement in temporal validation. *Conclusions*: AI-derived CVB parameters from preoperative CXR were independently associated with postoperative in-hospital MACE and provided modest incremental predictive information beyond routinely available clinical factors. Automated CVB analysis may provide additional cardiovascular risk information from a routinely obtained preoperative CXR without additional imaging or patient burden, although further external validation is required to establish its clinical utility.

## 1. Introduction

Chest radiography (CXR) remains one of the most commonly obtained imaging studies in perioperative practice, owing to its speed, low cost, and wide availability [1,2]. Despite its frequent use, routine preoperative CXR has limited incremental value for perioperative risk prediction and is generally recommended only in patients with specific clinical indications [3,4]. Interpretation of cardiovascular structures on CXR has traditionally relied on qualitative visual assessment or simple manual measurements, most notably the cardiothoracic (CT) ratio [5,6,7,8,9,10]. However, this approach reduces complex cardiomediastinal morphology to a single metric, does not account for age- or sex-related physiological variation, and may fail to detect subtle abnormalities in individual cardiomediastinal vascular borders (CVBs) [11].

The Automated Diagnosis of Cardiovascular abnormalities (ADC) model was developed to overcome these limitations [11,12]. Conventional CVB analysis has historically been subjective and lacked established normative reference ranges; the ADC model addresses both shortcomings by enabling automated, reproducible quantification of CVBs on posteroanterior CXR. The model delineates predefined CVBs, extracts standardized linear and angular measurements, and converts them into age- and sex-adjusted z-scores using previously validated reference data. In the original validation study, this framework demonstrated diagnostic utility across several cardiovascular disease groups and prognostic value in patients with coronary artery disease, suggesting that quantitative CXR phenotyping may provide clinically relevant structural information beyond the CT ratio alone.

Despite these advances, the potential role of AI-derived CVB analysis in perioperative risk stratification has not been established. In surgical patients, the preoperative cardiomediastinal configuration may reflect underlying cardiovascular comorbidity, chronic hemodynamic burden, and physiological reserve, all of which may influence early postoperative outcomes. We therefore hypothesized that quantitative CVB metrics automatically derived from preoperative CXR would be associated with postoperative major adverse cardiovascular events (MACE). Accordingly, this study aimed to evaluate the associations and discriminative performance of AI-derived CVB parameters for postoperative in-hospital MACE and to determine whether selected CVB parameters provide incremental predictive information beyond routinely available clinical factors.

## 2. Materials and Methods

### 2.1. Study Design and Population

This retrospective study was approved by the Institutional Review Board of the study center (IRB protocol number 2024-1517), and the requirement for informed consent was waived because of the retrospective design and use of de-identified data. The study was conducted in accordance with the principles of the Declaration of Helsinki.

Adult patients who underwent surgery under general anesthesia at a single tertiary academic medical center between 1 March 2019 and 28 February 2023 were screened for inclusion. Preoperative CXRs were routinely performed as part of standard surgical care at our institution. For each surgical episode, a preoperative CXR obtained within 6 months before surgery was considered eligible. When multiple eligible CXRs were available, the radiograph obtained closest to the date of surgery was used. To minimize duplication bias arising from multiple surgical episodes in the same patient, only the first (index) eligible surgical episode per patient was retained for analysis, and subsequent operations during the study period were excluded. Patients were also excluded if they were 19 years of age or younger, had no eligible preoperative CXR within 6 months before surgery, or had a CXR automatically rejected by the ADC software. The ADC software automatically excluded anteroposterior or lateral radiographs and images with inadequate positioning, severe artifacts, or poor lung area segmentation, including hyperinflation, hypoinflation, and asymmetric lung fields. Preoperative CXRs for which the ADC model failed to generate at least one CVB parameter were also excluded.

### 2.2. Clinical Variables and Outcome

Clinical data were extracted from the institutional electronic medical records and included demographic characteristics, comorbidities, American Society of Anesthesiologists (ASA) physical status, emergency surgery, and type of surgery. The primary outcome was postoperative in-hospital MACE, defined as a composite of cardiac arrest, new-onset atrial fibrillation, ventricular arrhythmia, demand myocardial ischemia (ST-T segment changes with associated symptoms), acute myocardial infarction, heart failure (excluding chronic and end-stage heart failure), and stroke. MACE was ascertained algorithmically from diagnoses recorded in the discharge summary using International Classification of Diseases, 10th Revision codes (ICD-10). Only diagnoses first recorded after surgery were considered eligible; diagnoses corresponding to the same code documented during any prior hospitalization or before surgery during the index admission were excluded to restrict ascertainment to new-onset postoperative events. Patients were followed from the date of surgery until hospital discharge, in-hospital death, or the occurrence of the first qualifying event, whichever occurred first.

### 2.3. CXR Analysis Using the ADC Model

**Figure 1:**
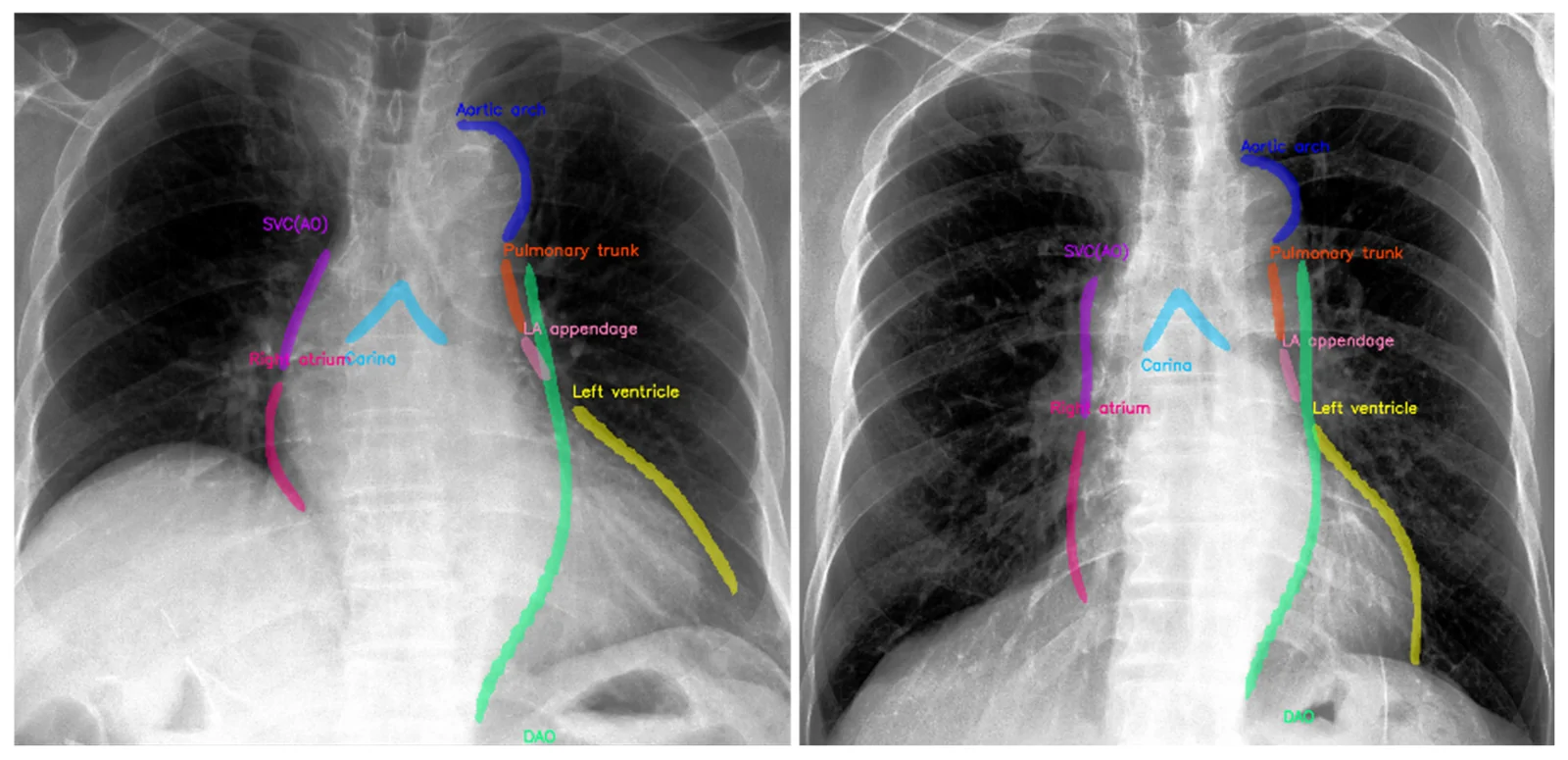
Representative preoperative posteroanterior CXRs demonstrating ADC-derived CVB measurements in elderly male patients with (**left**) and without (**right**) postoperative MACE. The ADC model automatically delineates predefined CVB parameters evaluated in this study.

Eligible preoperative CXRs were analyzed using the ADC model, a previously developed and externally validated deep learning-based algorithm for automated quantification of CVB parameters on posteroanterior CXRs [11,12]. The model automatically delineates predefined CVBs and extracts standardized linear and angular measurements without human intervention (Figure 1).

For each CXR, the ADC model quantified the following parameters:

- CT ratio: the maximal horizontal cardiac diameter divided by the maximal horizontal thoracic diameter
- Superior vena cava/ascending aorta (SVC/AO): the right upper CVB
- Right atrium (RA): the right lower CVB
- Left atrial (LA) appendage: the normally flat region between the pulmonary artery and the lower left CVB
- Left ventricle (LV): the lower left cardiomediastinal contour
- Aortic arch: the upper left laterally projecting contour First bullet;
- Descending aorta: the lateral contour extending inferiorly from the aortic arch
- Pulmonary trunk: the bulge beneath the aortic arch
- Carina angle: the angle formed between the lower borders of the right and left main bronchi. LA enlargement may widen the carinal angle

All measurements were standardized into age- and sex-adjusted z-scores using previously established reference ranges from the ADC study. The composite CVB z-score was derived in two steps. First, the age- and sex-adjusted z-scores of the nine individual CVB parameters were summed with equal weights for each patient. Second, the resulting sum was standardized within the study cohort to a mean of 0 and a standard deviation of 1. Thus, a one-unit increase in the composite CVB z-score represents a one-standard-deviation increase in the summed CVB z-scores within the study cohort. Equal weighting was used to avoid outcome-driven weighting and reduce the risk of overfitting. Patients with a missing value for any of the nine individual CVB parameters were excluded from calculation of the composite CVB z-score.

### 2.4. Statistical Analysis

Continuous variables are presented as mean ± standard deviation (SD) if normally distributed, or as median with interquartile range [Q1, Q3] if non-normally distributed, as assessed by the Shapiro-Wilk test. Categorical variables are presented as number (%). Baseline clinical characteristics and ADC-derived CVB measurements were compared between MACE and non-MACE groups using the Student’s t-test or Mann-Whitney U test for continuous variables, and the chi-square test or Fisher exact test for categorical variables, as appropriate. No formal sample size calculation was performed; all eligible patients during the study period were included to maximize statistical power. A complete-case approach was applied to ADC-derived CVB parameters, and no imputation was performed for missing CVB measurements; there were no missing data for the clinical covariates included in this study.

Associations between ADC-derived CVB parameters and postoperative in-hospital MACE were evaluated using logistic regression models. Univariable analyses were performed first, followed by multivariable analyses adjusted for prespecified clinical covariates. Multivariable models were adjusted for age, sex, ASA physical status, emergency surgery, and comorbidities directly related to MACE, including arrhythmia, ischemic heart disease, cerebrovascular disease, and chronic kidney disease. Results are reported as unadjusted odds ratios (OR) and adjusted OR, with 95% confidence intervals (CIs). ORs are presented per scale-unit increase in each continuous variable: 0.05 for CT ratio, 3 mm or degrees for raw measurements, and 1 unit for age- and sex-adjusted z-scores and the composite CVB z-score.

The prognostic performance of individual ADC-derived CVB parameters for postoperative in-hospital MACE was assessed using receiver operating characteristic (ROC) curve analysis. Area under the curve (AUC) with 95% CIs were calculated, and the optimal cutoff value for each parameter was determined by the maximum Youden index.

Several sensitivity analyses were performed to assess the robustness of the primary findings. First, the association and discrimination analyses were repeated after excluding patients who underwent cardiac surgery. Second, to evaluate the potential influence of the interval between CXR acquisition and surgery, analyses were repeated after restricting the cohort to patients with CXRs obtained within 90 days and within 30 days before surgery. Third, the analyses were repeated using a restricted cardiovascular endpoint comprising cardiac arrest, acute myocardial infarction, and stroke. For each sensitivity analysis, the same multivariable adjustment strategy and ROC analysis used in the primary analysis were applied.

To assess the incremental predictive value of CVB parameters beyond routinely available clinical information, three prediction models were constructed. Model 1 was a clinical reference model including age, sex, ASA physical status, emergency surgery, arrhythmia, ischemic heart disease, cerebrovascular disease, and chronic kidney disease. Model 2 extended Model 1 by adding the raw CT ratio, and Model 3 extended Model 1 by adding the composite CVB z-score. Discrimination was assessed using the AUC with 95% CIs, and AUCs of Models 2 and 3 were compared with that of Model 1 using DeLong’s test for correlated ROC curves. Incremental predictive performance was further assessed using the integrated discrimination improvement and net reclassification improvement. Temporal validation included assessment of discrimination and calibration, while bootstrap internal validation and decision curve analysis were used to evaluate model optimism and clinical utility, respectively.

All statistical analyses were performed using Python version 3.8.15 with scipy and scikit-learn packages. A two-sided *p* value < 0.05 was considered statistically significant.

## 3. Results

### 3.1. Study Population

A total of 213,808 surgical episodes were screened during the study period. After exclusion of patients 19 years of age or younger (*n* = 25,033), those without an eligible preoperative CXR within 6 months before surgery (*n* = 69,761), repeated operations beyond the index surgery for an individual patient (*n* = 16,481), and CXRs automatically rejected by the ADC software (*n* = 1002), 101,531 preoperative CXRs were included in the final analysis. The median interval between preoperative CXR and surgery was 17 days (IQR 2–67). Representative ADC-derived CVB measurements on preoperative CXRs are shown in Figure 1.

### 3.2. Baseline Clinical Characteristics and CVB Measurements

Postoperative in-hospital MACE occurred in 1655 patients (1.6%). The most frequent MACE component was new-onset atrial fibrillation (*n* = 763, 46.1% of patients with MACE), followed by heart failure (*n* = 442, 26.7%), stroke (*n* = 292, 17.6%), acute myocardial infarction (*n* = 80, 4.8%), demand ischemia (*n* = 77, 4.7%), and ventricular arrhythmia (*n* = 59, 3.6%). No cardiac arrest was identified using the prespecified ICD-10–based ascertainment algorithm. Because some patients experienced more than one qualifying event, the individual component counts were not mutually exclusive.

Table 1 summarizes the baseline clinical characteristics according to MACE occurrence. Patients with MACE were older (64.7 ± 12.5 vs. 54.7 ± 14.9 years; *p* < 0.001), more often male (57.5% vs. 39.6%; *p* < 0.001), and had a lower body mass index (23.8 ± 3.6 vs. 24.2 ± 3.8 kg/m^2^; *p* < 0.001). The MACE group had a higher prevalence of diabetes, hypertension, arrhythmia, ischemic heart disease, cerebrovascular disease, chronic kidney disease, and respiratory disease (*p* < 0.001 for all except respiratory disease, *p* = 0.003), as well as a higher ASA physical status and a higher frequency of emergency surgery (10.8% vs. 2.8%; *p* < 0.001).

Table 2 compares CVB measurements between groups. All ADC-derived CVB parameters were significantly greater in the MACE group than in the non-MACE group (*p* < 0.001 for all).

### 3.3. Association Between CVB Parameters and MACE

In univariable and multivariable logistic regression analyses (Table 3), all raw CXR-derived CVB parameters were associated with postoperative in-hospital MACE after adjustment for age, sex, ASA physical status, emergency surgery, and comorbidities including arrhythmia, ischemic heart disease, cerebrovascular disease, and chronic kidney disease. For CT ratio, the unadjusted OR was 2.15 per 0.05-unit increase (95% CI, 2.08–2.21; *p* < 0.001), and the adjusted OR was 1.64 (95% CI, 1.58–1.70; *p* < 0.001). For the remaining raw CVB parameters, adjusted ORs per 3-mm increase ranged from 1.04 (aortic arch; 95% CI, 1.02–1.06) to 1.16 (LA appendage; 95% CI, 1.14–1.17) (*p* < 0.001 for all).

When age- and sex-adjusted z-scores were analyzed, all CVB z-scores were associated with MACE in both univariable and multivariable models. For CT ratio z-score, the unadjusted OR was 2.56 (95% CI, 2.45–2.69; *p* < 0.001), and the adjusted OR was 1.85 (95% CI, 1.76–1.94; *p* < 0.001). For the remaining CVB z-scores, adjusted ORs ranged from 1.08 (aortic arch; 95% CI, 1.04–1.13) to 1.60 (LV; 95% CI, 1.53–1.67) (*p* < 0.001 for all). For the composite CVB z-score, the unadjusted OR was 2.60 (95% CI, 2.49–2.71; *p* < 0.001), and the adjusted OR was 1.88 (95% CI, 1.79–1.97; *p* < 0.001).

In a sensitivity analysis excluding patients who underwent cardiac surgery, the associations between CVB parameters and postoperative in-hospital MACE remained consistent. All raw CVB measurements and individual age- and sex-adjusted CVB z-scores remained significantly associated with MACE after multivariable adjustment (*p* < 0.001 for all). The composite CVB z-score was also independently associated with MACE (Supplementary Table S1).

Similar findings were observed when the analysis was restricted to patients with more recent preoperative imaging. Among patients with CXRs obtained within 90 days before surgery (*n* = 78,772), associations between CVB parameters and MACE were generally consistent with the primary analysis. Results were also largely preserved when the imaging window was restricted to 30 days (*n* = 64,545); all age- and sex-adjusted CVB z-scores, including the composite z-score, remained significantly associated with MACE, whereas the association for the raw SVC/AO measurement did not reach statistical significance (*p* = 0.056) (Supplementary Table S2).

In an additional sensitivity analysis using a restricted cardiovascular endpoint comprising cardiac arrest, acute myocardial infarction, and stroke, 369 patients experienced an event, although no cardiac arrest was identified using the prespecified ICD-10–based ascertainment algorithm. Associations with the restricted endpoint were generally consistent with the primary analysis, with all raw CVB measurements and all age- and sex-adjusted CVB z-scores, including the composite z-score, remaining significantly associated after multivariable adjustment (Supplementary Table S3).

### 3.4. Discriminative Performance of CVB Parameters for MACE

**Figure 2:**
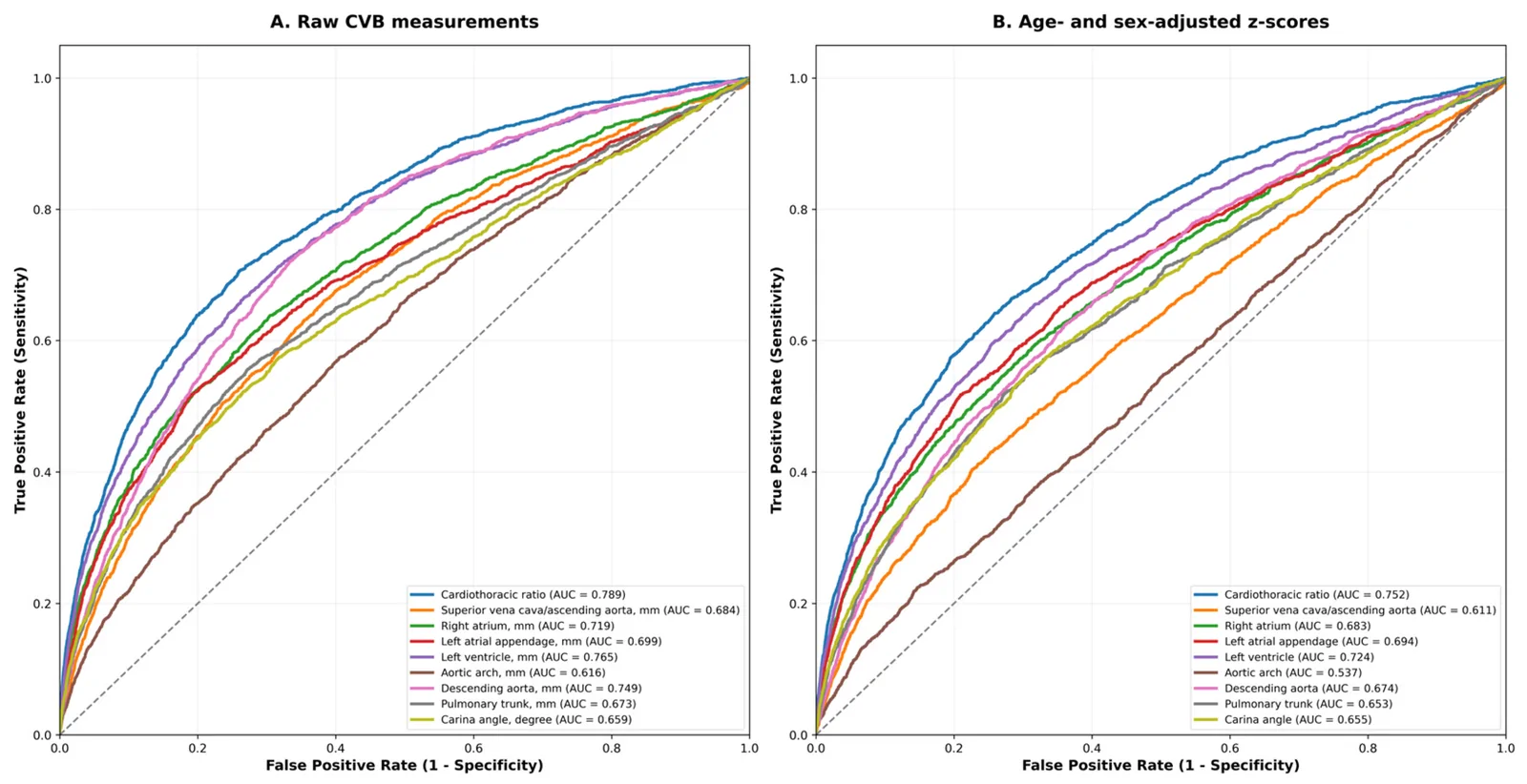
ROC curves of ADC-derived CVB parameters for postoperative MACE. (**A**) raw CVB parameter measurements; (**B**) CVB parameters standardized into age- and sex-adjusted z-scores using previously established reference ranges from the ADC study.

ROC analysis is shown in Figure 2 and Table 4. Among raw measurements, the AUC for CT ratio was 0.789 (95% CI, 0.777–0.803). AUCs for the remaining raw CVB parameters ranged from 0.616 (aortic arch; 95% CI, 0.601–0.631) to 0.765 (LV; 95% CI, 0.752–0.775). Optimal cutoffs for all parameters are provided in Table 4.

When standardized as age- and sex-adjusted z-scores, the AUC for CT ratio was 0.752 (95% CI, 0.742–0.766). AUCs for the remaining CVB z-scores ranged from 0.537 (aortic arch; 95% CI, 0.521–0.553) to 0.724 (LV; 95% CI, 0.706–0.735). The composite CVB z-score achieved an AUC of 0.762 (95% CI, 0.750–0.773).

In the sensitivity analysis excluding cardiac surgery, the discriminative performance of the CVB parameters remained similar to that observed in the overall cohort (Supplementary Table S1). Similar discrimination was observed when the analysis was restricted to CXRs obtained within 90 or 30 days before surgery; the AUCs for the raw CT ratio were 0.795 and 0.796, respectively, and those for the composite CVB z-score were 0.770 and 0.769, respectively (Supplementary Table S2). For the restricted cardiovascular endpoint, discrimination was lower than for the primary composite MACE outcome, although the overall pattern across CVB parameters was maintained; the AUCs were 0.731 for the raw CT ratio and 0.702 for the composite CVB z-score (Supplementary Table S3).

### 3.5. Incremental Predictive Performance of CVB Parameters

Adding either the raw CT ratio or the composite CVB z-score to the clinical reference model improved discrimination, increasing the AUC from 0.894 to 0.906 (*p* < 0.001 for both by DeLong test). The corresponding integrated discrimination improvement values were 0.0287 and 0.0279, respectively (both *p* < 0.001). This improvement was maintained in temporal validation, with AUCs of 0.909 for the model including the raw CT ratio and 0.907 for the model including the composite CVB z-score, compared with 0.894 for the clinical reference model. Calibration slopes were close to 1, and Brier scores decreased from 0.0167 to 0.0161 for both extended models (Supplementary Table S4 and Supplementary Figure S1). Bootstrap internal validation demonstrated minimal optimism in AUC (0.0006–0.0007), with optimism-corrected AUCs of 0.894, 0.905, and 0.905 for Models 1–3, respectively (Supplementary Table S5). Decision curve analysis showed limited separation among models at lower threshold probabilities but greater net benefit for the extended models at higher thresholds (Supplementary Figure S2).

## 4. Discussion

In this large-scale retrospective cohort study of 101,531 surgical patients, we demonstrated that all AI-derived CVB parameters were associated with postoperative in-hospital MACE. For raw CT ratio, the adjusted OR was 1.64, and the AUC was 0.789. The composite CVB z-score showed an adjusted OR of 1.88 and an AUC of 0.762. Adding either measure to the clinical reference model modestly improved discrimination with similar findings in temporal validation and sensitivity analyses. To our knowledge, this study represents the first large-scale investigation of AI-derived quantitative CVB phenotypes in relation to postoperative in-hospital MACE in a general surgical population.

Preoperative CXR is widely obtained in perioperative practice, and automated analysis may allow additional quantitative information to be extracted without additional imaging or patient burden. Prior CXR-based AI studies have primarily focused on direct outcome prediction from radiographic images. Weiss et al. developed a deep learning model that estimated 10-year cardiovascular risk from routine CXRs in an outpatient screening setting [13]. Raghu et al. similarly applied a deep learning model to preoperative CXRs and demonstrated performance comparable to established risk scores in cardiac surgery populations [14]. These studies differ from the present study in both population and analytic approach. Rather than training an AI model to predict postoperative outcomes directly from CXR images, the ADC model automatically identifies and quantifies predefined cardiomediastinal vascular structures, generating interpretable anatomical measurements and age- and sex-adjusted z-scores. We subsequently evaluated the associations of these AI-derived structural phenotypes with postoperative in-hospital MACE using multivariable statistical models. Accordingly, the present findings should be interpreted as evidence that automated CXR phenotyping provides quantitative structural information associated with postoperative cardiovascular events, rather than as validation of an AI-based MACE prediction model. Because the populations, outcomes, and modeling strategies differ substantially across studies, direct comparison of discriminatory performance is not appropriate.

A modest attenuation in discriminative performance was observed when raw measurements were replaced by age- and sex-adjusted z-scores, which may partly reflect the removal of age- and sex-related variation through standardization, as both are established cardiovascular risk factors [15]. The z-scores represent deviations from age- and sex-specific reference values, and because raw measurements and z-scores were modeled using different units, their OR magnitudes are not directly comparable. Nevertheless, CVB z-scores remained independently associated with MACE after multivariable adjustment. The composite CVB z-score did not improve discrimination beyond the simpler CT ratio, either alone or when added to the clinical reference model, suggesting substantial overlap in the prognostic information captured by the two measures. Moreover, because the composite score was re-standardized within the present cohort, its absolute values are cohort-dependent and would require recalibration before application to other populations.

Beyond these associations, CVB parameters provided incremental predictive information when added to routinely available clinical variables. Adding either the raw CT ratio or the composite CVB z-score to the clinical reference model increased the AUC from 0.894 to 0.906, with similar improvements in temporal validation. Incremental discrimination was also supported by significant integrated discrimination improvement values, and bootstrap validation demonstrated minimal optimism. Calibration was generally good. However, the absolute improvement in discrimination was modest, and decision curve analysis showed limited separation between models at lower threshold probabilities, although the extended models provided greater net benefit at higher thresholds. These findings suggest that automated CVB measurements provide additional prognostic information beyond conventional clinical variables, while their clinical utility for perioperative decision-making requires further evaluation.

The robustness of the primary associations was further supported by several sensitivity analyses. Similar findings were observed after exclusion of cardiac surgery and when analyses were restricted to CXRs obtained within 90 or 30 days before surgery. Associations also persisted when a more restricted cardiovascular endpoint was used, although discrimination was modestly lower than for the broader composite MACE outcome. These findings support the robustness of the observed associations across variations in surgical population, imaging interval, and outcome definition, while not eliminating the possibility of residual confounding or outcome misclassification.

Several potential explanations may underlie the observed associations between individual CVB parameters and MACE, although the present observational study cannot establish the underlying mechanisms. CT ratio enlargement and LV border widening may reflect increased cardiac mass and volume, which are associated with impaired hemodynamic reserve and heightened susceptibility to demand ischemia under the physiological stress of surgery [16]. LA appendage enlargement may be associated with elevated LA pressure and an atrial fibrillation substrate [17,18], which is the most common cardiac arrhythmia in the perioperative period [19]. Aortic arch and descending aortic enlargement may reflect systemic atherosclerotic burden and increased aortic stiffness, both of which are associated with adverse cardiovascular outcomes [20]. Pulmonary trunk widening may be associated with elevated pulmonary arterial pressure or right ventricular pressure overload, conditions that reduce right heart reserve during the hemodynamic challenges of surgery and anesthesia [21,22]. However, these radiographic findings are nonspecific and were not corroborated by echocardiographic, computed tomographic, or hemodynamic measurements in the present study. Therefore, the observed associations should be interpreted as relationships between radiographic structural phenotypes and postoperative cardiovascular events rather than evidence of specific pathophysiological mechanisms.

This study has several limitations. First, the retrospective, single-center design and the requirement for an eligible, technically acceptable preoperative CXR may have introduced selection bias and limit generalizability; external validation is therefore required. Second, postoperative MACE was ascertained using ICD-10 discharge coding without independent chart adjudication or prior validation of the ascertainment algorithm, and the heterogeneous composite outcome may therefore be subject to misclassification despite the consistent findings in the restricted-endpoint sensitivity analysis. Third, detailed surgical categories and procedure-specific cardiovascular risk were unavailable, leaving potential residual confounding despite consistent findings after excluding cardiac surgery. Variables required to reconstruct established perioperative risk scores were also unavailable, and direct comparison with published performance estimates was inappropriate because of differences in populations, outcomes, and validation settings. Fourth, although sensitivity analyses restricted to CXRs obtained within 90 and 30 days yielded similar findings, the 6-month eligibility window may still permit changes in cardiopulmonary status between imaging and surgery. Finally, the age- and sex-adjusted CVB z-scores did not account for body size, and the composite CVB z-score was re-standardized within the present cohort, limiting direct transferability without recalibration. Cutoff values derived in this cohort should therefore be considered exploratory.

## 5. Conclusions

Preoperative CXR is routinely obtained in surgical patients, yet its structural information has traditionally been underused beyond visual assessment or the CT ratio. Automated quantitative extraction of CVB parameters enables additional structural information to be obtained from existing CXRs without additional imaging or patient burden. AI-derived CVB parameters were independently associated with postoperative in-hospital MACE, and selected CVB measures provided modest incremental predictive information beyond routinely available clinical factors. Further multicenter external validation is needed to determine whether these findings translate into clinically useful perioperative risk stratification.

## Figures and Tables

**Table 1:** Baseline characteristics according to postoperative MACE occurrence.

	Total (*N* = 101,531)	MACE Presence (*n* = 1655)	MACE Absence (*n* = 99,876)	*p*-Value
Demographics				
Age, years	54.8 ± 14.9	64.7 ± 12.5	54.7 ± 14.9	<0.001
Male sex	40,505 (39.9%)	952 (57.5%)	39,553 (39.6%)	<0.001
Height, cm	162.2 ± 8.5	162.6 ± 9.5	162.2 ± 8.5	0.010
Weight, kg	63.9 ± 12.3	63.2 ± 12.6	63.9 ± 12.3	0.019
Body mass index, kg/m^2^	24.2 ± 3.8	23.8 ± 3.6	24.2 ± 3.8	<0.001
Comorbidities				
Diabetes	15,094 (14.9%)	311 (18.8%)	14,783 (14.8%)	<0.001
Hypertension	30,518 (30.1%)	574 (34.7%)	29,944 (30.0%)	<0.001
Arrhythmia	2253 (2.2%)	168 (10.1%)	2085 (2.1%)	<0.001
Ischemic heart disease	3353 (3.3%)	85 (5.1%)	3268 (3.3%)	<0.001
Cerebrovascular disease	2198 (2.1%)	105 (6.3%)	2093 (2.1%)	<0.001
Chronic kidney disease	1692 (1.7%)	59 (3.6%)	1633 (1.6%)	<0.001
Dyslipidemia	13,621 (13.4%)	154 (9.3%)	13,467 (13.5%)	<0.001
Respiratory disease	2954 (2.9%)	69 (4.2%)	2885 (2.9%)	0.003
Clinical and surgical variables				
ASA physical status				<0.001
I	8166 (8.0%)	2 (0.1%)	8164 (8.2%)	
II	77,414 (76.2%)	277 (16.7%)	77,137 (77.2%)	
III	14,572 (14.4%)	1086 (65.6%)	13,486 (13.5%)	
IV	1332 (1.3%)	279 (16.9%)	1053 (1.1%)	
V	47 (0.0%)	11 (0.7%)	36 (0.0%)	
Emergency	2991 (3.0%)	178 (10.8%)	2813 (2.8%)	<0.001
Cardiac surgery	4946 (4.9%)	81 (4.9%)	4865 (4.9%)	0.954

Values are mean ± standard deviation, or number with proportion (%). Abbreviations: MACE, major adverse cardiovascular events; ASA, American Society of Anesthesiologists.

**Table 2:** AI-derived CVB parameters according to postoperative MACE occurrence.

	Total (*N* = 101,531)	MACE Presence (*n* = 1655)	MACE Absence (*n* = 99,876)	*p*-Value
Raw CVB measurements				
CT ratio	0.48 ± 0.06	0.56 ± 0.08	0.48 ± 0.06	<0.001
SVC/AO, mm	29.5 ± 7.9	35.3 ± 10.4	29.4 ± 7.8	<0.001
Right atrium, mm	39.3 ± 8.9	47.9 ± 12.8	39.1 ± 8.8	<0.001
LA appendage, mm	47.6 ± 8.5	54.8 ± 11.3	47.5 ± 8.4	<0.001
Left ventricle, mm	85.8 ± 12.9	99.5 ± 14.9	85.6 ± 12.8	<0.001
Aortic arch, mm	37.7 ± 7.8	41.4 ± 9.5	37.7 ± 7.8	<0.001
Descending aorta, mm	35.0 ± 10.8	45.5 ± 12.0	34.9 ± 10.7	<0.001
Pulmonary trunk, mm	39.3 ± 7.5	44.6 ± 9.6	39.2 ± 7.4	<0.001
Carina angle, degree	74.4 ± 9.7	81.0 ± 12.6	74.3 ± 9.6	<0.001
Age- and sex-adjusted z-scores				
CT ratio	0.14 ± 1.08	1.20 ± 1.19	0.12 ± 1.06	<0.001
SVC/AO	0.23 ± 1.04	0.65 ± 1.23	0.22 ± 1.04	<0.001
Right atrium	0.11 ± 1.09	0.91 ± 1.37	0.10 ± 1.08	<0.001
LA appendage	0.21 ± 1.13	1.06 ± 1.33	0.19 ± 1.12	<0.001
Left ventricle	0.26 ± 1.17	1.28 ± 1.36	0.25 ± 1.15	<0.001
Aortic arch	0.02 ± 1.09	0.21 ± 1.23	0.02 ± 1.08	<0.001
Descending aorta	0.20 ± 1.07	0.86 ± 1.15	0.19 ± 1.06	<0.001
Pulmonary trunk	0.19 ± 1.11	0.84 ± 1.29	0.18 ± 1.11	<0.001
Carina angle	0.32 ± 1.06	0.95 ± 1.22	0.31 ± 1.06	<0.001
Composite z-score	0.00 ± 1.00	1.11 ± 1.23	−0.02 ± 0.99	<0.001

Values are mean ± standard deviation. Abbreviations: CVB, cardiomediastinal vascular border; MACE, major adverse cardiovascular events; CT, cardiothoracic; SVC/AO, superior vena cava/ascending aorta; LA, left atrial.

**Table 3:** Logistic regression analyses of CVB parameters for postoperative MACE.

	Scale	Unadjusted OR (95% CIs)	*p*-Value	Adjusted OR (95% CIs)	*p*-Value
Raw CVB measurements					
CT ratio	0.05	2.15 (2.08–2.21)	<0.001	1.64 (1.58–1.70)	<0.001
SVC/AO, mm	3	1.24 (1.22–1.26)	<0.001	1.09 (1.08–1.11)	<0.001
Right atrium, mm	3	1.28 (1.26–1.29)	<0.001	1.14 (1.12–1.16)	<0.001
LA appendage, mm	3	1.28 (1.27–1.31)	<0.001	1.16 (1.14–1.17)	<0.001
Left ventricle, mm	3	1.26 (1.24–1.27)	<0.001	1.14 (1.13–1.15)	<0.001
Aortic arch, mm	3	1.18 (1.16–1.2)	<0.001	1.04 (1.02–1.06)	<0.001
Descending aorta, mm	3	1.25 (1.24–1.27)	<0.001	1.12 (1.11–1.14)	<0.001
Pulmonary trunk, mm	3	1.29 (1.26–1.31)	<0.001	1.14 (1.12–1.16)	<0.001
Carina angle, degree	3	1.21 (1.19–1.23)	<0.001	1.12 (1.10–1.13)	<0.001
Age- and sex-adjusted z-scores					
CT ratio	1	2.56 (2.45–2.69)	<0.001	1.85 (1.76–1.94)	<0.001
SVC/AO	1	1.48 (1.41–1.55)	<0.001	1.27 (1.21–1.33)	<0.001
Right atrium	1	1.92 (1.84–2.01)	<0.001	1.48 (1.41–1.54)	<0.001
LA appendage	1	1.96 (1.88–2.05)	<0.001	1.51 (1.44–1.58)	<0.001
Left ventricle	1	2.13 (2.04–2.22)	<0.001	1.60 (1.53–1.67)	<0.001
Aortic arch	1	1.17 (1.12–1.22)	<0.001	1.08 (1.04–1.13)	<0.001
Descending aorta	1	1.79 (1.71–1.87)	<0.001	1.44 (1.37–1.51)	<0.001
Pulmonary trunk	1	1.70 (1.62–1.77)	<0.001	1.44 (1.37–1.51)	<0.001
Carina angle	1	1.78 (1.70–1.86)	<0.001	1.44 (1.37–1.51)	<0.001
Composite z-score	1	2.60 (2.49–2.71)	<0.001	1.88 (1.79–1.97)	<0.001

Abbreviations: CVB, cardiomediastinal vascular border; MACE, major adverse cardiovascular events; OR, odds ratio; CI, confidence interval; CT, cardiothoracic; SVC/AO, superior vena cava/ascending aorta; LA, left atrial.

**Table 4:** ROC analysis of AI-derived CVB parameters for postoperative MACE prediction.

	AUC (95% CIs)	Cut-Off Value	Sensitivity (%)	Specificity (%)
Raw CVB measurements				
CT ratio	0.789 (0.777–0.803)	0.52	70.6	74.1
SVC/AO, mm	0.684 (0.672–0.698)	30.7	66.1	61.7
Right atrium, mm	0.719 (0.707–0.730)	42.8	63.8	69.6
LA appendage, mm	0.699 (0.686–0.712)	54.5	51.8	80.9
Left ventricle, mm	0.765 (0.752–0.775)	93.1	66.6	73.3
Aortic arch, mm	0.616 (0.601–0.631)	39.0	57.4	59.5
Descending aorta, mm	0.749 (0.737–0.761)	38.8	72.2	66.5
Pulmonary trunk, mm	0.673 (0.661–0.689)	43.2	55.7	72.8
Carina angle, degree	0.659 (0.647–0.674)	78.4	57.3	68.4
Age- and sex-adjusted z-scores				
CT ratio	0.752 (0.742–0.766)	0.82	64.2	74.1
SVC/AO	0.611 (0.592–0.624)	1.00	40.4	77.3
Right atrium	0.683 (0.668–0.693)	0.61	59.2	68.4
LA appendage	0.694 (0.678–0.708)	1.10	51.8	79.0
Left ventricle	0.724 (0.706–0.735)	1.01	59.6	74.5
Aortic arch	0.537 (0.521–0.553)	1.15	22.4	85.3
Descending aorta	0.674 (0.662–0.688)	0.57	62.4	63.9
Pulmonary trunk	0.653 (0.638–0.667)	0.84	51.9	72.8
Carina angle	0.655 (0.642–0.671)	0.83	55.1	69.3
Composite z-score	0.762 (0.750–0.773)	0.64	64.9	76.1

Cutoff values were selected by the maximum Youden index in this cohort and are exploratory. Abbreviations: ROC, receiver operating characteristic; CVB, cardiomediastinal vascular border; MACE, major adverse cardiovascular events; AUC, area under the curve; CI, confidence interval; CT, cardiothoracic; SVC/AO, superior vena cava/ascending aorta; LA, left atrial.

## Data Availability

The data presented in this study are available on request from the corresponding author.

## Supplementary Materials

The following supporting information can be downloaded at: https://www.mdpi.com/article/10.3390/medicina62101897/s1, Table S1: Sensitivity analysis excluding cardiac surgery: associations and discriminative performance of CVB parameters for postoperative in-hospital MACE; Table S2: Sensitivity analysis restricted to CXRs obtained within 90 and 30 days before surgery: associations and discriminative performance of CVB parameters for postoperative in-hospital MACE; Table S3: Sensitivity analysis using a restricted cardiovascular endpoint com-prising cardiac arrest, acute myocardial infarction, and stroke: associations and discriminative performance of CVB parameters; Table S4: Incremental predictive performance and temporal validation of models for postoperative in-hospital MACE; Table S5: Reclassification and bootstrap internal validation of extended models for postoperative in-hospital MACE; Figure S1: Calibration of the clinical reference and extended models for postoperative in-hospital MACE in the temporal validation cohort; Figure S2: Decision curve analysis of the clinical reference and extended models for postoperative in-hospital MACE.

## Abbreviations

The following abbreviations are used in this manuscript:

ADC	Automated Diagnosis of Cardiovascular Abnormalities
ASA	American Society of Anesthesiologists
CT	Cardiothoracic (Ratio)
CVB	Cardiomediastinal Vascular Border
CXR	Chest Radiograph(s)
ICD-10	International Classification of Diseases, 10th Revision
LA	Left Atrial
LV	Left Ventricle
MACE	Major Adverse Cardiovascular Events
RA	Right Atrium
SVC/AO	Superior Vena Cava/Ascending Aorta
