# Supplementary material for: AI-Powered Preoperative Chest Radiograph-Derived Cardiovascular Border Indices and Risk of Postoperative Major Adverse Cardiovascular Events: A Large-Scale Retrospective Cohort Study
Source: Medicina (Kaunas). 2026 Sep 30;62(10):1897. doi: 10.3390/medicina62101897 (PMC13707251; doi:10.3390/medicina62101897)
Supplement: Supplementary file 1 [file medicina-62-01897-s001.zip › medicina-4517212-supplementary.pdf]

Supplementary Table S1. Sensitivity analysis excluding cardiac surgery: associations and discriminative performance of CVB parameters for postoperative in-hospital MACE

	Scale	adjusted OR (95% CI)	*P*-value	AUC (95% CI)
Raw CVB measurements				
CT ratio	0.05	1.62 (1.56–1.68)	< 0.001	0.790 (0.779–0.801)
SVC/AO, mm	3	1.09 (1.07–1.11)	< 0.001	0.684 (0.670–0.696)
Right atrium, mm	3	1.13 (1.12–1.15)	< 0.001	0.718 (0.702–0.728)
LA appendage, mm	3	1.15 (1.14–1.17)	< 0.001	0.702 (0.685–0.718)
Left ventricle, mm	3	1.14 (1.12–1.15)	< 0.001	0.765 (0.754–0.777)
Aortic arch, mm	3	1.04 (1.02–1.06)	< 0.001	0.617 (0.603–0.630)
Descending aorta, mm	3	1.12 (1.10–1.14)	< 0.001	0.750 (0.739–0.764)
Pulmonary trunk, mm	3	1.14 (1.12–1.16)	< 0.001	0.676 (0.663–0.689)
Carina angle, degree	3	1.12 (1.10–1.13)	< 0.001	0.663 (0.646–0.677)
Age- and sex-adjusted z-scores				
CT ratio	1	1.82 (1.73–1.91)	< 0.001	0.752 (0.739–0.765)
SVC/AO	1	1.26 (1.20–1.32)	< 0.001	0.610 (0.593–0.625)
Right atrium	1	1.45 (1.39–1.52)	< 0.001	0.681 (0.670–0.695)
LA appendage	1	1.50 (1.44–1.57)	< 0.001	0.696 (0.683–0.710)
Left ventricle	1	1.58 (1.51–1.65)	< 0.001	0.724 (0.709–0.737)
Aortic arch	1	1.08 (1.04–1.13)	< 0.001	0.538 (0.520–0.553)
Descending aorta	1	1.43 (1.36–1.50)	< 0.001	0.674 (0.660–0.684)
Pulmonary trunk	1	1.38 (1.32–1.45)	< 0.001	0.656 (0.640–0.670)
Carina angle	1	1.44 (1.37–1.51)	< 0.001	0.659 (0.645–0.673)
Composite z-score	1	1.86 (1.77–1.95)	< 0.001	0.763 (0.752–0.779)

Abbreviations: CVB, cardiomediastinal vascular border; MACE, major adverse cardiovascular events; OR, odds ratio; CI, confidence interval; AUC, area under the receiver operating characteristic curve; CT, cardiothoracic; SVC/AO, superior vena cava/ascending aorta; LA, left atrial

Supplementary Table S2. Sensitivity analysis restricted to CXRs obtained within 90 and 30 days before surgery: associations and discriminative performance of CVB parameters for postoperative in-hospital MACE

		**CXR within 90 days**			**CXR within 30 days**		
	**Scale**	**adjusted OR (95% CI)**	***P*-value**	**AUC (95% CI)**	**adjusted OR (95% CI)**	***P*-value**	**AUC (95% CI)**
Raw CVB measurements							
CT ratio	0.05	1.32 (1.22–1.44)	< 0.001	0.795 (0.783–0.807)	1.33 (1.22–1.45)	< 0.001	0.796 (0.787–0.808)
SVC/AO, mm	3	1.04 (1.01–1.08)	0.022	0.687 (0.673–0.703)	1.04 (1.00–1.08)	0.056	0.684 (0.672–0.698)
Right atrium, mm	3	1.04 (1.01–1.08)	0.014	0.727 (0.715–0.743)	1.04 (1.00–1.07)	0.032	0.725 (0.712–0.738)
LA appendage, mm	3	1.09 (1.06–1.13)	< 0.001	0.708 (0.695–0.723)	1.09 (1.06–1.13)	< 0.001	0.710 (0.695–0.727)
Left ventricle, mm	3	1.07 (1.04–1.10)	< 0.001	0.775 (0.763–0.784)	1.07 (1.04–1.10)	< 0.001	0.776 (0.761–0.790)
Aortic arch, mm	3	1.10 (1.05–1.14)	< 0.001	0.616 (0.601–0.632)	1.10 (1.06–1.15)	< 0.001	0.615 (0.600–0.633)
Descending aorta, mm	3	1.11 (1.07–1.15)	< 0.001	0.751 (0.737–0.760)	1.11 (1.07–1.15)	< 0.001	0.749 (0.736–0.762)
Pulmonary trunk, mm	3	1.12 (1.07–1.16)	< 0.001	0.681 (0.667–0.696)	1.12 (1.07–1.17)	< 0.001	0.681 (0.665–0.697)
Carina angle, degree	3	1.06 (1.03–1.09)	< 0.001	0.662 (0.645–0.677)	1.06 (1.03–1.10)	< 0.001	0.663 (0.645–0.678)
Age- and sex-adjusted z-scores							
CT ratio	1	1.85 (1.75–1.96)	< 0.001	0.762 (0.749–0.774)	1.85 (1.76–1.96)	< 0.001	0.762 (0.749–0.776)
SVC/AO	1	1.25 (1.19–1.32)	< 0.001	0.613 (0.599–0.627)	1.27 (1.20–1.33)	< 0.001	0.611 (0.594–0.629)
Right atrium	1	1.46 (1.39–1.53)	< 0.001	0.689 (0.675–0.702)	1.47 (1.40–1.54)	< 0.001	0.688 (0.675–0.703)
LA appendage	1	1.51 (1.44–1.59)	< 0.001	0.702 (0.685–0.715)	1.52 (1.44–1.59)	< 0.001	0.703 (0.687–0.719)

Left ventricle	1	1.61 (1.53–1.69)	< 0.001	0.733 (0.722–0.747)	1.61 (1.54–1.69)	< 0.001	0.733 (0.716–0.745)
Aortic arch	1	1.08 (1.02–1.13)	0.004	0.535 (0.520–0.550)	1.08 (1.03–1.13)	0.002	0.534 (0.518–0.548)
Descending aorta	1	1.43 (1.35–1.51)	< 0.001	0.682 (0.666–0.698)	1.44 (1.37–1.52)	< 0.001	0.681 (0.667–0.695)
Pulmonary trunk	1	1.38 (1.32–1.46)	< 0.001	0.660 (0.642–0.678)	1.39 (1.32–1.46)	< 0.001	0.660 (0.641–0.673)
Carina angle	1	1.41 (1.34–1.49)	< 0.001	0.660 (0.645–0.676)	1.42 (1.35–1.50)	< 0.001	0.660 (0.647–0.678)
Composite z-score	1	1.86 (1.76–1.97)	< 0.001	0.770 (0.756–0.782)	1.88 (1.78–1.98)	< 0.001	0.769 (0.755–0.781)

Supplementary Table S3. Sensitivity analysis using a restricted cardiovascular endpoint comprising cardiac arrest, acute myocardial infarction, and stroke: associations and discriminative performance of CVB parameters

	**Scale**	**adjusted OR (95% CI)**	***P*-value**	**AUC (95% CI)**
Raw CVB measurements				
CT ratio	0.05	1.32 (1.23–1.42)	< 0.001	0.731 (0.703–0.755)
SVC/AO, mm	3	1.05 (1.01–1.08)	0.009	0.647 (0.616–0.674)
Right atrium, mm	3	1.05 (1.02–1.08)	< 0.001	0.652 (0.626–0.683)
LA appendage, mm	3	1.09 (1.06–1.12)	< 0.001	0.646 (0.613–0.673)
Left ventricle, mm	3	1.06 (1.04–1.09)	< 0.001	0.701 (0.673–0.727)
Aortic arch, mm	3	1.09 (1.05–1.13)	< 0.001	0.638 (0.610–0.666)
Descending aorta, mm	3	1.11 (1.08–1.14)	< 0.001	0.714 (0.688–0.741)
Pulmonary trunk, mm	3	1.11 (1.07–1.15)	< 0.001	0.647 (0.617–0.676)
Carina angle, degree	3	1.06 (1.03–1.09)	0.009	0.616 (0.588–0.655)
Age- and sex-adjusted z-scores				
CT ratio	1	1.39 (1.27–1.53)	< 0.001	0.695 (0.665–0.722)
SVC/AO	1	1.10 (1.00–1.21)	0.041	0.573 (0.549–0.605)
Right atrium	1	1.15 (1.06–1.26)	0.002	0.609 (0.581–0.634)
LA appendage	1	1.25 (1.15–1.37)	< 0.001	0.637 (0.601–0.661)
Left ventricle	1	1.21 (1.11–1.32)	< 0.001	0.652 (0.622–0.681)
Aortic arch	1	1.22 (1.12–1.33)	< 0.001	0.574 (0.545–0.603)
Descending aorta	1	1.34 (1.22–1.48)	< 0.001	0.641 (0.614–0.671)
Pulmonary trunk	1	1.27 (1.17–1.39)	< 0.001	0.625 (0.590–0.663)
Carina angle	1	1.21 (1.10–1.33)	< 0.001	0.610 (0.586–0.641)
Composite z-score	1	1.45 (1.32–1.60)	< 0.001	0.702 (0.672–0.729)

No cardiac arrest was identified using the prespecified ICD-10–based ascertainment algorithm in this study cohort. Abbreviations: CVB, cardiomediastinal vascular border; MACE, major adverse cardiovascular events; OR, odds ratio; CI, confidence interval; AUC, area under the receiver operating characteristic curve; CT, cardiothoracic; SVC/AO, superior vena cava/ascending aorta; LA, left atrial

Supplementary Table S4. Incremental predictive performance and temporal validation of models for postoperative in-hospital MACE

**Model**	**AUC (95% CI)**	**ΔAUC vs Model 1**	**P-value†**	**Temporal validation AUC (95% CI)**	**Calibration slope**	**Calibration intercept**	**Brier score**
Model 1: Clinical reference	0.894 (0.887–0.902)	—	—	0.894 (0.881–0.907)	1.034	0.284	0.0167
Model 2: Clinical reference + raw CT ratio	0.906 (0.899–0.913)	+0.0119	<0.001	0.909 (0.897–0.921)	1.027	0.268	0.0161
Model 3: Clinical reference + composite CVB z-score	0.906 (0.898–0.914)	+0.0117	<0.001	0.907 (0.894–0.920)	1.026	0.238	0.0161

Model 1 included age, sex, ASA physical status, emergency surgery, arrhythmia, ischemic heart disease, cerebrovascular disease, and chronic kidney disease. Model 2 additionally included the raw CT ratio, and Model 3 additionally included the composite CVB z-score. Temporal validation was performed using the 2022–2023 cohort. †Comparison with Model 1 using DeLong's test.

Abbreviations: AUC, area under the receiver operating characteristic curve; CI, confidence interval; CT, cardiothoracic; CVB, cardiomediastinal vascular border; MACE, major adverse cardiovascular events.

Supplementary Table S5. Reclassification and bootstrap internal validation of extended models for postoperative in-hospital MACE

Comparison / Model	IDI (95% CI)	*P*-value	NRI, total	NRI, events	NRI, non-events	Apparent AUC	Mean optimism	Optimism-corrected AUC
Model 2 vs Model 1	0.0287 (0.0231–0.0353)	<0.001	0.551	0.203	0.348	—	—	—
Model 3 vs Model 1	0.0279 (0.0224–0.0343)	<0.001	0.569	0.231	0.337	—	—	—
Model 1	—	—	—	—	—	0.894	0.0006	0.894
Model 2	—	—	—	—	—	0.906	0.0007	0.905
Model 3	—	—	—	—	—	0.906	0.0007	0.905

Model definitions are provided in Supplementary Table 4. Bootstrap internal validation was used to estimate optimism in model discrimination.

Abbreviations: AUC, area under the receiver operating characteristic curve; CI, confidence interval; IDI, integrated discrimination improvement; MACE, major adverse cardiovascular events; NRI, net reclassification improvement.

Supplementary Figure S1. Calibration of the clinical reference and extended models for postoperative in-hospital MACE in the temporal validation cohort.

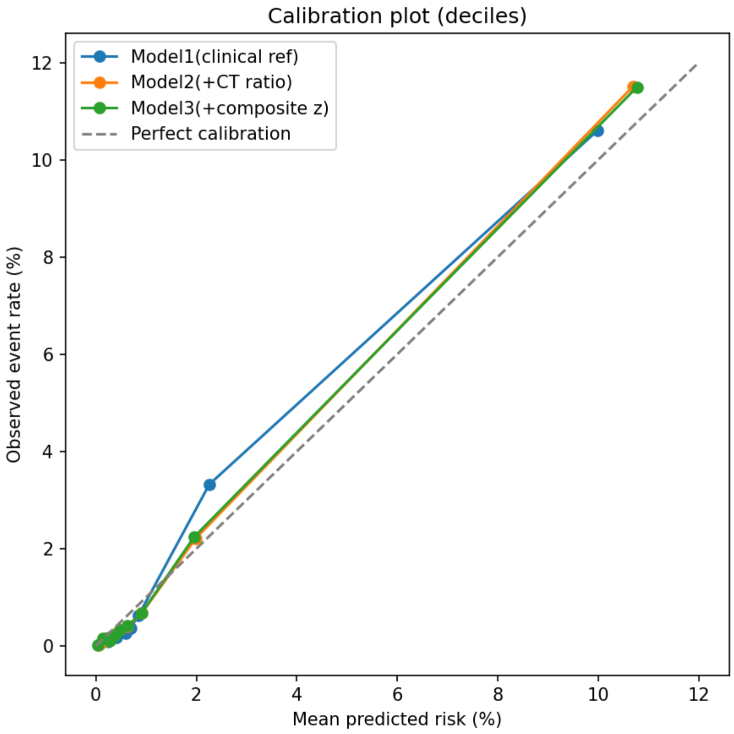

Observed event rates are plotted against mean predicted probabilities across deciles of predicted risk. The dashed diagonal line represents perfect calibration. Model 1 is the clinical reference model; Model 2 additionally includes the raw CT ratio; and Model 3 additionally includes the composite CVB z-score.

Abbreviations: CT, cardiothoracic; CVB, cardiomediastinal vascular border; MACE, major adverse cardiovascular events.

Supplementary Figure S2. Decision curve analysis of the clinical reference and extended models for postoperative in-hospital MACE.

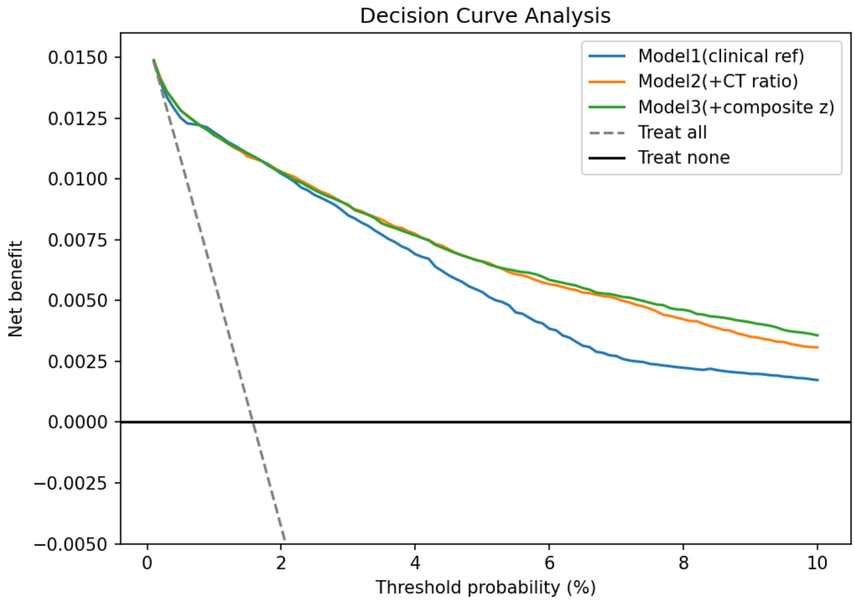

Net benefit is shown across threshold probabilities from 0% to 10% for the clinical reference model (Model 1), the clinical reference model plus raw CT ratio (Model 2), and the clinical reference model plus composite CVB z-score (Model 3). The treat-all and treat-none strategies are shown for comparison.

Abbreviations: CT, cardiothoracic; CVB, cardiomediastinal vascular border; MACE, major adverse cardiovascular events.
